# Supplementary figures and images for: A refined approach for evaluating small datasets via binary classification using machine learning
Source: PLoS One. 2024 May 21;19(5):e0301276. doi: 10.1371/journal.pone.0301276 (PMC11108166; doi:10.1371/journal.pone.0301276)

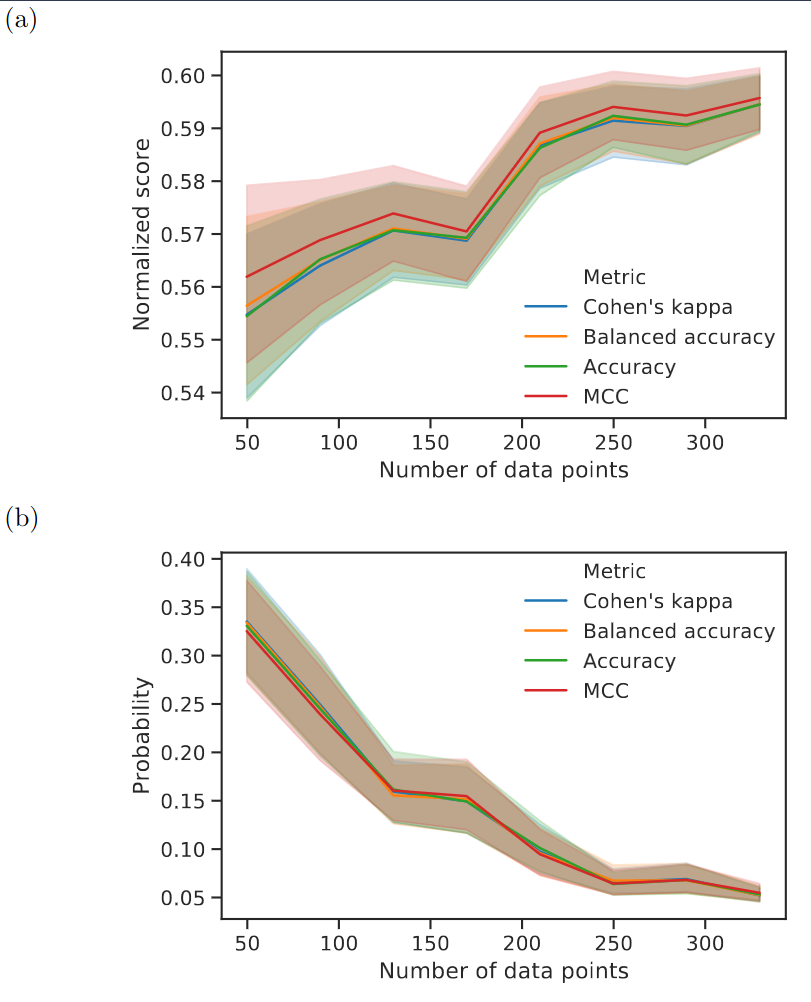

Supplement: S1 Fig — The scores of MCC and Cohen’s Kappa were normalised to the same range of values from 0 to 1 as the other two metrics. The coloured area represents the 95% confidence interval calculated by bootstraping over all datasets. (TIF) [file pone.0301276.s001.tif]
