# Supplementary material for: A refined approach for evaluating small datasets via binary classification using machine learning
Source: PLoS One. 2024 May 21;19(5):e0301276. doi: 10.1371/journal.pone.0301276 (PMC11108166; doi:10.1371/journal.pone.0301276)
Supplement: S1 Table — In this table, the advantages and disadvantages of ACC, BA, κ, and F1 Score are presented. (PDF) [file pone.0301276.s002.pdf]

S1 Table. Overview of the characteristics of different metrics. In this table, the advantages and disadvantages of accuracy (ACC), balanced accuracy (BA), Cohen's Kappa, and F_1 Score are presented.

	Advantages	Disadvantages
Accuracy	Intuitive: Ratio between the number of correctly classified and the number of all sample [1]<br>Works in multiclass case [1]<br>Generates reliable results for balanced datasets [1]<br>Includes all four categories (TP, FP, TN, FN)	Overestimates classifier performance on majority class for unbalanced datasets and can produce misleading results [1]
Balanced Accuracy	Symmetric with regard to the type of class [2]	A high value close to 1 does not imply high TPR, TNR, PPV and NPV [3]
Cohen's Kappa	Corrected for chance agreement [4]<br>Includes all 4 categories (TP, FP, TN, FN)	Not suited for imbalanced datasets [4]<br>Can be misleading, particularly when true positives and true negatives are zero [5]<br>Can result in higher values for worse classification results [4]<br>Kappa paradox [6]
F_1 Score	More relevant than Matthew's correlation coefficient, if positive data instances are more important than negative ones [3]	Results may be misleading for imbalanced datasets [1]<br>Independent of number of true negatives [1]<br>Varies for class swapping [1]

References

1. Chicco D, Jurman G. The advantages of the Matthews correlation coefficient (MCC) over F1 score and accuracy in binary classification evaluation. BMC Genomics. 2020;21(1):6. doi:10.1186/s12864-019-6413-7.

2. Brodersen KH, Ong CS, Stephan KE, Buhmann JM. The Balanced Accuracy and Its Posterior Distribution. In: 2010 20th International Conference on Pattern Recognition; 2010. p. 3121–3124.

3. Chicco D, Tötsch N, Jurman G. The Matthews correlation coefficient (MCC) is more reliable than balanced accuracy, bookmaker informedness, and markedness in two-class confusion matrix evaluation. BioData Mining. 2021;14(1):13. doi:10.1186/s13040-021-00244-z.

4. Delgado R, Tibau XA. Why Cohen's Kappa should be avoided as performance measure in classification. PLOS ONE. 2019;14(9):e0222916. doi:10.1371/journal.pone.0222916.

5. Chicco D, Warrens MJ, Jurman G. The Matthews Correlation Coefficient (MCC) is More Informative Than Cohen's Kappa and Brier Score in Binary Classification Assessment. IEEE Access. 2021;9:78368–78381. doi:10.1109/ACCESS.2021.3084050.

6. Feinstein AR, Cicchetti DV. High agreement but low Kappa: I. the problems of two paradoxes. Journal of Clinical Epidemiology. 1990;43(6):543–549. doi:https://doi.org/10.1016/0895-4356(90)90158-L.
