# Supplementary material for: A refined approach for evaluating small datasets via binary classification using machine learning
Source: PLoS One. 2024 May 21;19(5):e0301276. doi: 10.1371/journal.pone.0301276 (PMC11108166; doi:10.1371/journal.pone.0301276)
Supplement: S2 Table — In this table, the advantages and disadvantages of precision/positive predictive value (PPV), recall, AUC and MCC are presented. (PDF) [file pone.0301276.s003.pdf]

S2 Table. Overview of the characteristics of different metrics. In this table, the advantages and disadvantages of precision/positive predictive value (PPV), recall, receiver operating characteristic curve (ROC) and Matthews correlation coefficient (MCC) are presented.

	Advantages	Disadvantages
**MCC**	Suited if the positive class and the negative class have the same importance in the analysis [1]	If the prevalence in the dataset does not reflect the prevalence in the population, the metric will not return the performance of the classifier for the population of interest [1]
	High value close to 1 implies high TPR, TNR, PPV, and NPV [1,2]	Dependence on prevalence leads to non-transferability of results across datasets [1]
	Value of 0 implies classifier performance is equal to random guessing [1]	Not a robust indicator of how similar a classifier is to random guessing [1]
	Suited for imbalance classes [3]	
	Considers all elements of the binary confusion matrix [3,4]	
	Invariant for class swapping [3]	
**PPV**	Intuitive: Accuracy of the positive predictions [5,6]	If the prevalence in the dataset does not reflect the prevalence in the population, the metric will not return the performance of the classifier for the population of interest [1]
		Dependence on prevalence leads to non-transferability of results across datasets [1]
**Recall**	Measures how well positive instances are classified by the model [2]	Influenced by the ratio of data classes [2]
		Often overestimates the model performance [2]
**ROC**	Evaluates the performance of a discriminant across varying decision thresholds [7]	It is possible to obtain similar AUC values for datasets with opposite positive/negative ratios [2]
		Large inaccuracies in the estimated validation parameters associated with ROC analysis for small sample sizes [7]

References

1. Chicco D, Tötsch N, Jurman G. The Matthews correlation coefficient (MCC) is more reliable than balanced accuracy, bookmaker informedness, and markedness in two-class confusion matrix evaluation. BioData Mining. 2021;14(1):13. doi:10.1186/s13040-021-00244-z.

2. Brown JB. Classifiers and their metrics quantified. Molecular Informatics. 2018;37(1-2):1700127. doi:10.1002/minf.201700127.

3. Chicco D, Jurman G. The advantages of the Matthews correlation coefficient (MCC) over F1 score and accuracy in binary classification evaluation. BMC Genomics. 2020;21(1):6. doi:10.1186/s12864-019-6413-7.

4. Chicco D, Warrens MJ, Jurman G. The Matthews Correlation Coefficient (MCC) is More Informative Than Cohen's Kappa and Brier Score in Binary Classification Assessment. IEEE Access. 2021;9:78368–78381. doi:10.1109/ACCESS.2021.3084050.

5. Géron A. Hands-on machine learning with Scikit-Learn, Keras, and TensorFlow: concepts, tools, and techniques to build intelligent systems. 2nd ed. Sebastopol, CA: O'Reilly Media, Inc; 2019.

6. Saito T, Rehmsmeier M. The Precision-Recall Plot Is More Informative than the ROC Plot When Evaluating Binary Classifiers on Imbalanced Datasets. PLOS ONE. 2015;10(3):e0118432. doi:10.1371/journal.pone.0118432.

7. Hanczar B, Hua J, Sima C, Weinstein J, Bittner M, Dougherty ER. Small-sample precision of ROC-related estimates. Bioinformatics. 2010;26(6):822–830. doi:10.1093/bioinformatics/btq037.
