# Supplementary material for: A refined approach for evaluating small datasets via binary classification using machine learning
Source: PLoS One. 2024 May 21;19(5):e0301276. doi: 10.1371/journal.pone.0301276 (PMC11108166; doi:10.1371/journal.pone.0301276)
Supplement: S4 Table — (PDF) [file pone.0301276.s005.pdf]

S4 Table. Probabilities of the ACC, recall, F_1-Score, and MCC for rnCV on a random subsets of the MNIST and BCWD datasets.

Datapoints	Points	ACC	F_1	MCC
MNIST	250	0.02	0.02	0.02
MNIST	50	0.02	0.02	0.02
MNIST	25	0.16	1.00	1.00
BCWD	250	0.02	0.02	0.02
BCWD	50	0.02	0.02	0.02
BCWD	25	0.02	0.02	0.02
