# Supplementary material for: A refined approach for evaluating small datasets via binary classification using machine learning
Source: PLoS One. 2024 May 21;19(5):e0301276. doi: 10.1371/journal.pone.0301276 (PMC11108166; doi:10.1371/journal.pone.0301276)
Supplement: S5 Table — (PDF) [file pone.0301276.s006.pdf]

S5 Table. Confusion matrices of the ACC, F_1-Score, and MCC for rnCV on a random subsets of the MNIST and BCWD datasets.

Dataset	Points	ACC	F_1	MCC
MNIST	515	$\begin{pmatrix} 41.8 & 1.0 \\ 0.5 & 6.7 \end{pmatrix}$	$\begin{pmatrix} 41.7 & 1.1 \\ 0.5 & 6.7 \end{pmatrix}$	$\begin{pmatrix} 41.8 & 1.0 \\ 0.5 & 6.7 \end{pmatrix}$
MNIST	50	$\begin{pmatrix} 8.6 & 0.2 \\ 0.3 & 0.9 \end{pmatrix}$	$\begin{pmatrix} 8.1 & 0.7 \\ 0.2 & 1.0 \end{pmatrix}$	$\begin{pmatrix} 8.1 & 0.7 \\ 0.2 & 1.0 \end{pmatrix}$
MNIST	25	$\begin{pmatrix} 1.3 & 3.0 \\ 0.0 & 0.7 \end{pmatrix}$	$\begin{pmatrix} 1.2 & 2.2 \\ 0.0 & 0.4 \end{pmatrix}$	$\begin{pmatrix} 1.6 & 2.8 \\ 0.0 & 0.5 \end{pmatrix}$
BCWD	515	$\begin{pmatrix} 68.5 & 2.9 \\ 3.5 & 38.9 \end{pmatrix}$	$\begin{pmatrix} 68.4 & 3.0 \\ 3.4 & 39.0 \end{pmatrix}$	$\begin{pmatrix} 68.4 & 3.0 \\ 3.4 & 39.0 \end{pmatrix}$
BCWD	50	$\begin{pmatrix} 6.8 & 0.2 \\ 0.2 & 2.8 \end{pmatrix}$	$\begin{pmatrix} 6.8 & 0.16 \\ 0.2 & 2.8 \end{pmatrix}$	$\begin{pmatrix} 6.8 & 0.2 \\ 0.2 & 2.8 \end{pmatrix}$
BCWD	25	$\begin{pmatrix} 2.5 & 0.3 \\ 0.4 & 1.8 \end{pmatrix}$	$\begin{pmatrix} 2.5 & 0.3 \\ 0.3 & 1.9 \end{pmatrix}$	$\begin{pmatrix} 2.5 & 0.3 \\ 0.4 & 1.8 \end{pmatrix}$
