# Supplementary material for: A refined approach for evaluating small datasets via binary classification using machine learning
Source: PLoS One. 2024 May 21;19(5):e0301276. doi: 10.1371/journal.pone.0301276 (PMC11108166; doi:10.1371/journal.pone.0301276)
Supplement: S6 Table — (PDF) [file pone.0301276.s007.pdf]

S6 Table. Scores of the MCC for rnCV missing either the hyperparameter tuning, the feature selection or both on a random subsets of the MNIST and BCWD dataset.

Dataset	Points	full rnCV	missing feature selection	missing hyperparameter tuning	missing feature selection and hyperparameter tuning
MNIST	515	0.89	0.97	0.89	0.96
MNIST	50	0.68	0.97	0.69	1.00
MNIST	25	0.00	0.38	0.00	0.38
BCWD	515	0.88	0.88	0.87	0.89
BCWD	50	0.92	0.83	0.90	0.90
BCWD	25	0.76	0.49	0.72	0.61
