# Supplementary material for: A refined approach for evaluating small datasets via binary classification using machine learning
Source: PLoS One. 2024 May 21;19(5):e0301276. doi: 10.1371/journal.pone.0301276 (PMC11108166; doi:10.1371/journal.pone.0301276)
Supplement: S7 Table — (PDF) [file pone.0301276.s008.pdf]

S7 Table. Probabilities of the MCC for rnCV missing either the hyperparameter tuning, the feature selection or both on a random subsets of the MNIST dataset.

Points	full	missing feature selection	missing hyper parameter tuning	missing feature selection and hyper parameter tuning
515	0.02	0.02	0.02	0.02
50	0.02	0.02	0.02	0.02
25	1.00	0.02	1.00	0.02
