# Supplementary material for: A refined approach for evaluating small datasets via binary classification using machine learning
Source: PLoS One. 2024 May 21;19(5):e0301276. doi: 10.1371/journal.pone.0301276 (PMC11108166; doi:10.1371/journal.pone.0301276)
Supplement: Attachment — Submitted filename: PONE-D-23-36197.pdf [file pone.0301276.s009.pdf]

PLOS ONE

A refined approach for evaluating small datasets via binary classification using machine learning

--Manuscript Draft--

**Manuscript Number:**	PONE-D-23-36197
**Article Type:**	Research Article
**Full Title:**	A refined approach for evaluating small datasets via binary classification using machine learning
**Short Title:**	A refined approach for evaluating small datasets via binary classification using machine learning
**Corresponding Author:**	Steffen Steinert<br>Ludwig-Maximilians-Universitat Munchen<br>Munich, GERMANY
**Keywords:**	machine learning, evaluation methods, small datasets, education research
**Abstract:**	Classical statistical analysis of data can be complemented or replaced with data analysis based on machine learning. However, in certain disciplines, such as education research, studies are frequently limited to small datasets, which raises several questions regarding biases and coincidentally positive results. In this study, we present a refined approach for evaluating the performance of a binary classification based on machine learning for small datasets. The approach includes a non-parametric permutation test as a method to quantify the probability of the results generalising to new data. Furthermore, we found that a repeated nested cross-validation is almost free of biases and yields reliable results that are only slightly dependent on chance. Considering the advantages of several evaluation metrics, we suggest a combination of more than one metric to train and evaluate machine learning classifiers. In the specific case that both classes are equally important, the Matthews correlation coefficient exhibits the lowest bias and chance for coincidentally good results. The results indicate that it is essential to avoid several biases when analysing small datasets using machine learning.
**Order of Authors:**	Steffen Steinert
	Verena Ruf
	David Dzsotjan
	Nicolas Großmann
	Albrecht Schmidt
	Jochen Kuhn
	Stefan Küchemann
**Additional Information:**	
**Question**	**Response**
**Financial Disclosure**<br><br>Enter a financial disclosure statement that describes the sources of funding for the work included in this submission. Review the submission guidelines for detailed requirements. View published research articles from *PLOS ONE* for specific examples.	The author(s) received no specific funding for this work.

Powered by Editorial Manager® and ProduXion Manager® from Aries Systems Corporation

This statement is required for submission and **will appear in the published article** if the submission is accepted. Please make sure it is accurate.<br><br>**Funded studies**<br>Enter a statement with the following details:<br>• Initials of the authors who received each award<br>• Grant numbers awarded to each author<br>• The full name of each funder<br>• URL of each funder website<br>• Did the sponsors or funders play any role in the study design, data collection and analysis, decision to publish, or preparation of the manuscript?<br><br>Did you receive funding for this work?	
**Competing Interests**<br><br>Use the instructions below to enter a competing interest statement for this submission. On behalf of all authors, disclose any competing interests that could be perceived to bias this work—acknowledging all financial support and any other relevant financial or non-financial competing interests.<br><br>This statement is required for submission and **will appear in the published article** if the submission is accepted. Please make sure it is accurate and that any funding sources listed in your Funding Information later in the submission form are also declared in your Financial Disclosure statement.<br><br>View published research articles from *PLOS ONE* for specific examples.	The authors have declared that no competing interests exist.

Powered by Editorial Manager® and ProduXion Manager® from Aries Systems Corporation

**NO authors have competing interests**<br><br>Enter: *The authors have declared that no competing interests exist.*<br><br>**Authors with competing interests**<br><br>Enter competing interest details beginning with this statement:<br><br>*I have read the journal's policy and the authors of this manuscript have the following competing interests: [insert competing interests here]*<br><br>* typeset	
**Ethics Statement**<br><br>Enter an ethics statement for this submission. This statement is required if the study involved:<br><br>• Human participants<br>• Human specimens or tissue<br>• Vertebrate animals or cephalopods<br>• Vertebrate embryos or tissues<br>• Field research<br><br>Write "N/A" if the submission does not require an ethics statement.<br><br>General guidance is provided below. Consult the submission guidelines for detailed instructions. **Make sure that all information entered here is included in the Methods section of the manuscript.**	N/A

**Format for specific study types**<br><br>**Human Subject Research (involving human participants and/or tissue)**<br>• Give the name of the institutional review board or ethics committee that approved the study<br>• Include the approval number and/or a statement indicating approval of this research<br>• Indicate the form of consent obtained (written/oral) or the reason that consent was not obtained (e.g. the data were analyzed anonymously)<br><br>**Animal Research (involving vertebrate animals, embryos or tissues)**<br>• Provide the name of the Institutional Animal Care and Use Committee (IACUC) or other relevant ethics board that reviewed the study protocol, and indicate whether they approved this research or granted a formal waiver of ethical approval<br>• Include an approval number if one was obtained<br>• If the study involved *non-human primates*, add *additional details* about animal welfare and steps taken to ameliorate suffering<br>• If anesthesia, euthanasia, or any kind of animal sacrifice is part of the study, include briefly which substances and/or methods were applied<br><br>**Field Research**<br><br>Include the following details if this study involves the collection of plant, animal, or other materials from a natural setting:<br>• Field permit number<br>• Name of the institution or relevant body that granted permission	
**Data Availability**<br><br>Authors are required to make all data underlying the findings described fully available, without restriction, and from the time of publication. PLOS allows rare exceptions to address legal and ethical concerns. See the PLOS Data Policy and FAQ for detailed information.	Yes - all data are fully available without restriction

Powered by Editorial Manager® and ProduXion Manager® from Aries Systems Corporation

A Data Availability Statement describing where the data can be found is required at submission. Your answers to this question constitute the Data Availability Statement and **will be published in the article**, if accepted.<br><br>**Important:** Stating 'data available on request from the author' is not sufficient. If your data are only available upon request, select 'No' for the first question and explain your exceptional situation in the text box.<br><br>Do the authors confirm that all data underlying the findings described in their manuscript are fully available without restriction?	
**Describe where the data may be found in full sentences. If you are copying our sample text, replace any instances of XXX with the appropriate details.**<br><br>• If the data are **held or will be held in a public repository**, include URLs, accession numbers or DOIs. If this information will only be available after acceptance, indicate this by ticking the box below. For example: *All XXX files are available from the XXX database (accession number(s) XXX, XXX.)*.<br>• If the data are all contained **within the manuscript and/or Supporting Information files**, enter the following: *All relevant data are within the manuscript and its Supporting Information files.*<br>• If neither of these applies but you are able to provide **details of access elsewhere**, with or without limitations, please do so. For example:<br><br>*Data cannot be shared publicly because of [XXX]. Data are available from the XXX Institutional Data Access / Ethics Committee (contact via XXX) for researchers who meet the criteria for access to confidential data.*<br><br>*The data underlying the results presented in the study are available from (include the name of the third party*	The Python implementation of the methodology developed and used in the study as well as the code to produce the results, is available under https://osf.io/8rkjb/?view_only=4352e24595d34cb29b763b71b018766d.

and contact information or URL).<br>• This text is appropriate if the data are owned by a third party and authors do not have permission to share the data.<br><br>* typeset	
Additional data availability information:	

Manuscript Click here to access/download;Manuscript;manuscript.pdf

A refined approach for evaluating small datasets via binary classification using machine learning

Steffen Steinert[1,2]*, Verena Ruf[1], David Dzsotjan[1], Nicolas Großmann[3], Albrecht Schmidt[4], Jochen Kuhn[1], Stefan Küchemann[1],

1 Chair of Physics Education, Ludwig-Maximilians-Universität München (LMU Munich), Geschwister-Scholl-Platz 1, 80539 Munich, Germany
2 Department of Electrical and Computer Engineering, RPTU Kaiserslautern-Landau, Erwin-Schrödinger-Str. 46, Kaiserslautern, 67663, Germany
3 Smart Data & Knowledge Services, German Research Center for Artificial Intelligence, Trippstadter Straße 122, Kaiserslautern, 67663, Germany
4 Human-Centered Ubiquitous Media, Ludwig-Maximilians-Universität München (LMU Munich), Frauenlobstraße 7a, Munich, 80337, Germany

* steinert.steffen@physik.uni-muenchen.de

Abstract

Classical statistical analysis of data can be complemented or replaced with data analysis based on machine learning. However, in certain disciplines, such as education research, studies are frequently limited to small datasets, which raises several questions regarding biases and coincidentally positive results. In this study, we present a refined approach for evaluating the performance of a binary classification based on machine learning for small datasets. The approach includes a non-parametric permutation test as a method to quantify the probability of the results generalising to new data. Furthermore, we found that a repeated nested cross-validation is almost free of biases and yields reliable results that are only slightly dependent on chance. Considering the advantages of several evaluation metrics, we suggest a combination of more than one metric to train and evaluate machine learning classifiers. In the specific case that both classes are equally important, the Matthews correlation coefficient exhibits the lowest bias and chance for coincidentally good results. The results indicate that it is essential to avoid several biases when analysing small datasets using machine learning.

Introduction

In recent decades, machine learning (ML) has become one of the most widely used tools for data analysis, as it significantly broadens classical statistical methods for analysing large datasets [1]—for example, proteins (e.g. [2]) or educational datasets (e.g. [3]). However, the analysis of small datasets with ML is not straightforward and can be subject to biases and methodological errors. This may lead to results that are not generalizable to other datasets and, therefore, are of limited use [4, 5].

For binary ML-based classification, different algorithms can be combined with various validation methods and performance metrics, such as accuracy [6]. The performance metrics also differ in terms of their sensitivity to different features of the data. For example, accuracy does not take into account imbalances in the dataset: for highly imbalanced datasets, one might obtain very high accuracy even though the

algorithm trivially predicts only the same class [7]. Furthermore, each validation method has different properties, therefore, they are not equally suitable for different types of dataset and some methods may produce highly variable or biased results [5]. For example, the train/test split method has a large variance for small datasets [8]. Moreover, a strong negative correlation between the number of data points and high reported accuracy was found in an evaluation of the results of several studies on autism [5]. This correlation is rather unexpected for ML algorithms and might be an indication of an underlying bias. Typically, the classification error decreases as the number of data points increases [9].

One approach to avoid overoptimistic evaluation of datasets is nested cross-validation (nCV) [10, 11]. In this method, an inner cross-validation loop is used to estimate the model performance for each hyperparameter combination, and an outer cross-validation loop is used to estimate the generalisation performance of the selected hyperparameters. However, repeating this method several times with the same data yields different results. This makes it necessary to repeat this method with different cross-validation (CV) splits [12].

Furthermore, standard methods such as train/test splitting or nCV do not provide information on the probability that the trained algorithm will randomly perform well in the validation process [13]. During the evaluation of the classifier performance, there may be inaccuracies that lead to an overly optimistic estimation of the prediction quality, which can have crucial social and medical consequences, such as those related to the diagnosis of COVID-19 from CXR or CT images [4].

In classical statistics, one of the most commonly used methods for hypothesis testing is the determination of the p-value [14]. The p-value indicates the probability that a null hypothesis—for example, that a treatment in an intervention group has no effect compared to a control group—is true. Below a certain threshold, such as 5%, the null hypothesis is rejected.

In ML, the determination of a probability comparable to the p-value is not well established, although it can contain valuable information regarding the probability of randomly high performance metrics. Although similar evaluation methods exist [15], they are usually not applied in practice [13]. Particularly for small datasets, which are common in education research, an estimated good prediction quality might also be due to chance [13]. This can happen if the features and the target variables are independent—that is, there is no difference between classes [15]—or if the classifier was not able to exploit the dependency in the data [16]. It is important to quantify a probability for this null hypothesis to be true, because in this case, the results obtained from a high probability dataset would not be generalizable to new data.

In this paper, we determine the probability of a null hypothesis for ML using a non-parametric permutation test [15, 17], analogous to the p-value in classical statistics, and highlight its importance for computation with small datasets. We demonstrate the relevance of repeated nested cross-validation (rnCV) in combination with robust performance metrics for binary classification.

Overall, we combine the results of several previous studies (e.g. [5, 6, 12, 18–21]) with our own experimental results on both a synthetically generated and a real educational dataset to demonstrate crucial factors in ML-based classification of small datasets. We conclude by introducing a method that is as independent of randomness and bias as possible and enables us to obtain a probability for the null hypothesis.

Materials and methods

Evaluated data

Several self-generated datasets as well as a real-world educational dataset are examined. Both predictable and non-predictable random datasets are generated to investigate the dependence of the prediction quality and the probability of the null hypothesis on the metric and the evaluation method used. To generate the unpredictable data with features that have the same distributions for each class, balanced datasets consisting of two classes are created. First, the first class consisting of half of the data points is generated, and then the features are generated so that their distribution corresponds to a Gaussian distribution with a standard deviation of 1 and a mean of 0. The same is done to generate the second class, and then all data points are shuffled. To generate the balanced datasets with differences among the class features, the procedure is analogous, except that the features of the second class are drawn from a Gaussian distribution with standard deviation 1 and mean 0.2. datasets with and without differences between classes are created with the following dimensions: To find the dependence of the score on the number of data points, datasets with 10 features and a number of data points between 25 and 45 are generated, with a step size of 5 data points. To cover a larger range, we also generate datasets with 10 features and a number of data points between 50 and 330, where each dataset has 40 more data points than the previous one. We choose this step size to find a compromise between computation time and resolution. In addition, to determine the mean and variance of the scores between the different datasets for different numbers of data points and features, 100 datasets of each dimensionality are generated and analysed. Of course, it would be advisable to analyse more datasets to obtain a more stable mean of the scores, but a balance has to be established between a sufficient number of datasets and the computational time required.

The CESAR dataset [22] is used to demonstrate the evaluation method on a real dataset and to investigate the dependence of the results on the metric in practice. This is a multi-part educational science dataset containing, among other things, the results of a test of representational ability on vector field plots and field lines [23], the results of a concept test of electromagnetism, as well as demographic data of the students. This test was administered online using the Unipark survey software [24]. A total of 515 students from the University of Saarland, the University of Kaiserslautern and the Swiss Federal Institute of Technology Zurich participated in the study and are included in the dataset. For the ML, only the 12 questions of the representational competence test, the completion time, and the self-assessment of the data quality were used. The column names of the features used are given in parentheses: duration (duration), data quality (DQ) and answers to questions 1–12 (repko_1–repko_12) with questions 4 and 5 split into four (4a–4d) and three (5a–5c) subquestions. In this paper, the responses to question 5b are predicted using the responses to the remaining items of the representational competency test, the test duration, and the data quality rating. To simulate real data collection and smaller datasets, the order of the data points is randomised and the first part of the resulting dataset is evaluated up to 25 data points, up to 50 data points, and up to the full size of 515 data points. We do not examine dataset sizes between 50 and 515 data points because 50 data points on this dataset already performs almost as well as the full data, and we do not expect to see much improvement. We do not examine dataset sizes below 25 data points because the performance is already not good for 25 data points. For dataset sizes between 25 and 50 data points, we believe that no additional insight would be gained, as the larger datasets contain the entirety of the smaller datasets.

Preprocessing

We did not eliminate outliers for the CESAR data because it uses the self-assessment of data quality, which can be learned by the algorithm if it really has an impact on the predictions. In addition, the evaluation of this dataset is only exemplary. Therefore, we did not focus on optimising the prediction. Since some of the data used have widely varying ranges of values, the data must be normalised or standardised. In this case, the data is standardised because we do not eliminate the outliers and the influence of the outliers is much smaller with standardisation than with normalisation [7]. In addition, the SVM algorithm used does not rely on a fixed range of values between 0 and 1. Since the answers to the questions are categories that are only coded by numbers, but have no further meaning (e.g. larger numbers are better), all columns except those for self-assessment of data quality and processing time were one-hot encoded (Supplementary section Training of the algorithm). Of course, the self-generated data do not need to be cleaned of outliers, and the normalisation of these data would not be necessary, but the influence of the metrics and the method should be studied as close to practice as possible. Therefore, the data is standardised so that a possible influence of this procedure is included in the results and so that realistic results can be obtained.

Feature selection

No feature selection was performed for the generated datasets, as they are generated so that all features have the same importance. For the CESAR dataset, the features were ranked using mutual information (see Supplementary section Training of the algorithm) and the most important features were used, treating the number of features to be selected as a hyperparameter to be adjusted. The mutual information implementation for categorical data from scikit-learn [25] was used. This method was selected because it is rather simple, fast, easy to implement, and—most importantly—non-parametric, which is sufficient to demonstrate the influence of feature selection. If the prediction quality is to be optimised and the choice of features is important, other more sophisticated methods are recommended (see the Supplementary section Training of the algorithm).

Handling class imbalances

For the self-generated datasets, there is no need to use a method for evaluating imbalanced datasets, as the data are generated in a balanced manner. For the CESAR dataset, we decided to use random oversampling because it is a simple, easy-to-understand method that is sufficient for the demonstration. However, random oversampling has a few disadvantages compared to more sophisticated methods (see the Supplementary section Training of the algorithm), so we generally recommend using alternatives as mentioned in section Training of the algorithm (e.g. SMOTE).

Hyperparameter tuning

For both the generated and CESAR datasets, the best hyperparameters for each dataset are searched for during training. This is done using the GridSearchCV function of scikit-learn [25], which attempts all possible combinations of the given parameters and selects the one with the highest prediction quality. To determine the prediction quality, the function uses cross-validation; in this case, we chose to use a stratified 5-CV, as it still yields good results but takes less time to compute than a 10-CV. The parameter space under investigation is spanned by the following parameters ‘C’: 0.1, 1, 10; ‘gamma’: 0.1, ‘scale’, ‘auto’; ‘kernel’: ‘linear’,‘rbf’,‘poly’,‘sigmoid’; ‘n_features_to_select’:

Fig 1. Structure of the nested cross-validation method in this study. It consists of an outer CV to evaluate the performance of the trained ML model and an inner CV to find the optimal (hyper)parameters and features of the model. In the innermost loop, feature selection, standardisation, and oversampling are applied to the training subset of the inner CV before passing the data to the SVM classifier. The combination of hyperparameters that achieves the highest score in the inner CV is used to retrain the model on the outer CV training data and predict its test data. The averaged results of all iterations of the outer CV are returned as the result.

10, 15, 20, 25, 30. Here, 'C' is the regularisation parameter, which controls the importance of misclassifications. 'gamma' controls the width of the bell-shaped curve of the RBF kernel, and the value 'scale' sets gamma to the inverse of the product of the number of features and their variance. The value 'auto' sets the gamma to the inverse of the number of features. The parameter 'n_features_to_select' controls how many features must be selected during feature selection. 'kernel" controls the SVM kernel used, where 'linear' assumes linear separability of classes, 'rbf' uses the RBF kernel, and 'sigmoid' uses the sigmoid kernel. (see Supplementary section Training of the algorithm). This is a simple method, which we consider sufficient in our case, since it is not our goal to achieve the best possible prediction quality. However, if the goal is to optimise the result, it may be worthwhile to use an automated hyperparameter optimisation (see Supplementary Section Training of the algorithm).

Determination of the probability of the null hypothesis

In this paper, we use an empirical, non-parametric permutation test (see the Supplementary section Comparison of Classification Accuracy to Chance Level) to test whether two groups (classifier performance on original data and on data with randomly permuted labels) are equal (null hypothesis), because for this test the dataset does not need to fulfil any assumptions regarding the statistical properties. In this manner, the presented approach is more transferable to various small datasets that may not meet the statistical requirements of a parametric test. We believe that the computational time required for the test is still acceptable for small datasets and the methods studied here. The test randomly permutes labels and reassigns them to other attributes, thereby breaking the link between labels and attributes. In this work, only 25 permutations are performed, and the performance of the ML algorithm is evaluated using both the rnCV and nCV methods. For the evaluation of the CESAR dataset without feature selection, we use 50 permutations to resolve the difference in the returned probability between the datasets comprising 515 data points and 50 data points. For the evaluation of the CESAR dataset with feature selection, we use only 25 permutations because we treat the number of features to be used as a hyperparameter, which makes the computation time much higher than without feature selection. In fact, 1000 or more permutations are required for an effective approximation of the probability (see Supplementary section Comparison of Classification Accuracy to Chance Level). However, the focus of this work is on the qualitative behaviour and the demonstration of the evaluation methodology and, therefore, fewer permutations are used for reasons of computational time. For each permutation and each dataset, an rnCV is performed with five repetitions as well as five nested CVs with different random seeds. This is done to estimate the influence of randomness, to obtain a mean that is less dependent on randomness, and to examine the difference between using repeated nested cross-validation and simple nested cross-validation in permutation testing. The same permutations are used to calculate the probability of the rnCV as those used to calculate the nCVs, so we use their results to calculate the results of the rnCV. In

addition, this allows for good comparability of results, as differences are entirely due to the calculation method and not due to chance.

Metrics

Both the training and the evaluation of the prediction quality of a dataset are always performed with the same metric. We study the following metrics: F_1, recall, precision, accuracy, balanced accuracy, Cohen's kappa, area under the receiver operating characteristic curve, and the MCC. We selected these metrics because they are commonly used in the literature and there are several papers that address their advantages and disadvantages (see Supplementary section Metrics).

Validation methods

The predictive quality of a model is evaluated for each dataset using both an rnCV and an nCV.

Nested cross-validation Depending on what you want to do with your data (e.g. use feature selection or not), the structure of the nCV used varies. The structure in our case is depicted in Figure 1. To avoid overly optimistic performance estimates and to increase comparability, particularly for asymmetric metrics, the minority class of the full dataset is always selected as the positive class. The outermost *stratified n-CV* divides the full dataset into n equal parts with class proportions that reflect the class proportions of the full dataset. Each of these parts is used once as test data to determine the prediction quality of the model trained in the following steps. To estimate the prediction quality, we choose n = 10 for the outer stratified n-fold CV, as it yields results with low bias and variance (see the Supplementary section Training of the algorithm). The other n-1 parts are passed to the *GridsearchCV* function of scikit-learn [25], which systematically uses all *m possible combinations of the given hyperparameters* (see section Hyperparameter tuning) and divides the training dataset passed from the outer n-CV into k parts with equal class ratios in another inner *stratified k-CV*. Each of these k parts is used once to test the prediction quality of the model created in the following steps, and the other k-1 parts are used to train the model. As a compromise between computational time and variance and bias of the results, we choose k=5 in the inner stratified k-fold CV for model selection. Thereafter, the training dataset of the inner CV is passed to a *pipeline*, which *selects features* based only on this training dataset (see section Feature selection), then *standardises* the data (see section Preprocessing), balances the class ratios by *random oversampling* (see section Handling class imbalances), and finally passes the processed training data to the actual *ML algorithm*. For the ML algorithm, we chose a support vector machine (SVM) because it is widely used and can handle small datasets. The SVM trained on these k-1 parts is then applied to the last part of the inner CV, which is kept for testing; using a metric such as the MCC, the *prediction quality score* is determined by comparing the predictions with the true values. After all k parts have been used once as a test dataset, the *k scores* of the prediction qualities are averaged and thus the prediction *score of the k-CV* is determined. This is done for all m combinations of hyperparameters. From the resulting *m scores*, the combination with the highest score is selected. The selected model is *refitted* to the full training dataset of the n-CV and used to predict the corresponding test dataset. After each of the n parts is used as a test dataset, the obtained *n scores* are averaged and thus the final *score of the nested CV* is obtained.

Repeated nested cross-validation The nCV method described above is repeated five times with different random seeds and the results are averaged to obtain the result

of the rnCV.

Results

Influence of the number of permutations on the result of the permutation test

We determined the probability that the null hypothesis is true for balanced synthetic datasets using a permutation test using repeated nested cross-validation (rnCV) and the Matthews correlation coefficient (MCC) (Fig. 2). At a sample size of 50 data points, the probability is between 0.30 and 0.33 for each number of permutations ($N = 25, 50, 75, 100$). The average probabilities decay with an increasing number of data points, and they cross a probability of 0.05 at approximately 230 data points ($N = 100$), at 235 data points ($N = 75$), and 240 data points ($N = 50$). The examined range up to 330 data points is not sufficient for 25 permutations to attain an average probability of less than 5%.

Fig 2. Dependence of the probability obtained with the permutation test using the MCC on the number of data points for different numbers of permutations. The probability obtained is depicted as a function of the number of data points for 25 (orange), 50 (blue), 75 (red), and 100 permutations (green). The coloured area represents the 95% confidence interval calculated over all datasets using bootstrapping—that is, it is derived from the results of repeated resampling of the population. The dotted grey line indicates a probability of 5%.

Comparison of metrics

A comparison of three common evaluation metrics reveals that the the average MCC increases slightly with increasing number of data points (Fig. 3), while the probability obtained by the permutation test decreases (Fig. 3). The MCC has a lower probability of the null hypothesis being true than the F_1 score and the recall for all data points. Although the increase in the F_1 score with more data points is greater than the increase in the MCC, there is a slower decrease in its probability. If we examine the dependence of the normalised recall score on size of the dataset (Fig. 3), the recall decreases as the number of data points increases. However, the recall shows values above 0.65, which is at the upper end of its value range. Despite the decrease in recall, the probability obtained by the permutation test (Fig. 3) nevertheless declines when there is a larger number of data points. The probability of recall is higher than that for the other metrics for all numbers of data points. Comparing MCC, Cohen's kappa, accuracy, and balanced accuracy (Supplementary Fig. S1 Fig), the trends are very similar and there are only very small deviations among them. As the amount of data increases, the 95% confidence interval decreases for all metrics, but this decrease is most evident for the MCC.

Fig 3. (a) Dependence of the normalised prediction score and (b) the probabilities of the corresponding permutation tests on the number of data points using MCC, F_1 and recall. The MCC score was normalised to the same range of values from 0 to 1 as the other two metrics. The coloured area represents the 95% confidence interval calculated by bootstrapping over all datasets.

Comparison of nCV and rnCV

To understand the influence of repetitions of the nested cross-validation, we averaged the results of all equally sized balanced synthetic datasets for each number of data points. We used five repetitions within the rnCV to determine the MCC and the probability and obtained the results for MCC and probability by averaging the results of five nCVs. This means that we used the same predictions to calculate the results for the nCVs and that of the rnCV; thus, only the method of calculation differs and, therefore, the results for this comparison are exact.

It must be noted that the score of the rnCV (see Fig. 4) is identical to the mean score of the nCVs. However, the probabilities of the null hypothesis being true are consistently higher for the nCV than for the rnCV (see Fig. 4).

Fig 4. Mean value of (a) the MCC and (b) the probability of 5 nCV versus the MCC of an rnCV consisting of five replicates. The red line represents the bisector for clarity and the connecting line between the data points is only a guide for the eye.

CESAR dataset

To quantify the impact of this procedure on a real dataset, we determined different metrics and the probability that the null hypothesis is true for the publicly available educational dataset, CESAR [22] (Tables 1, 2 and Supplementary Tables S3 Table). The CESAR project studies the conceptual understanding of electromagnetism supported by augmented reality experiments. In a pilot study, a survey with 12 items of a concept test and 13 items of a representational competence test was deployed. The CESAR dataset includes scores of 515 university students in concept and representational competence tests [26]. Here, we applied this procedure to predict students' performance in item 5b (see section Evaluated data) of these tests based on the score in the other items. The probabilities were calculated for 25 and 50 random data points of the dataset as well as for the full dataset of 515 points. In this manner, we effectively simulate results of smaller real datasets with comparable data quality. Tables 1 and 2 present the results of an evaluation without feature selection. Supplementary Tables S3 Table and S4 Table in the appendix present the results of an evaluation with feature selection.

For the full dataset and for 50 randomly selected data points, both with and without feature selection, all metrics are well above the expected value of a random data prediction (see Table 1 and Supplementary Table S3 Table) and the permutation tests indicate that the results are statistically significant—that is, $p < 0.05$ (see Table 2 and Supplementary Table S4 Table). Since the evaluations with feature selection were performed with only half of the permutations (for computational reasons), the smallest possible probability is approximately twice as high as in the evaluations without feature selection. For 25 data points, both with and without feature selection, the metrics are barely above the expected value of chance; the probabilities range from 7.8% to 38.8% and are, thus, not statistically significant.

In general, for the dataset with 25 data points, the feature selection significantly reduces the probabilities for all metrics apart from the accuracy, balanced accuracy, and recall. This effect cannot be observed for the datasets with 50 and 515 points, since they already have a very high probability with the number of permutations we used, and the resolution does not allow the representation of smaller changes.

Apart from this, the feature selection degrades both Cohen's κ and the F_1 score for the 25 point dataset, but significantly reduces the probability that the null hypothesis is true. The scores and probabilities for the full dataset and the dataset with 50 data points are similar, even though the full dataset has over 10 times as many data points.

The permutation test without feature selection provides the best probability of 0.02 for 50 permutations for the complete dataset for all metrics and the second best probability of 0.04 for the dataset consisting of 50 data points. In addition, the permutation test with feature selection yields the best probability of 0.04 for 25 permutations for the full dataset as well as for the dataset consisting of 50 data points for all metrics. Only the probability of the F_1 score for 50 data points is approximately 16%. Except for the F_1 score for the full dataset, feature selection improves the score for the full dataset as well as for the dataset consisting of 50 data points. For 25 data points, the score mostly remains the same or becomes slightly worse, with the probability remaining similar.

Table 1. Scores of the accuracy (ACC), balanced accuracy (BA), precision, recall, F_1-Score, MCC, the area under ROC curve (AUC), and Cohen's κ without feature selection for repeated nested cross-validation on a random subsets of the CESAR dataset.

Points	ACC	BA	Precision	Recall	F_1	MCC	AUC	κ
515	0.69	0.67	0.86	0.81	0.63	0.35	0.73	0.35
50	0.68	0.63	0.55	0.94	0.48	0.30	0.70	0.24
25	0.60	0.58	0.4	1.0	0.52	0.15	0.53	0.14

Table 2. Probabilities without feature selection for repeated nested cross-validation on random subsets of data.

Points	ACC	BA	Precision	Recall	F_1	MCC	AUC	κ
515	0.02	0.02	0.02	0.12	0.02	0.02	0.02	0.02
50	0.04	0.04	0.04	0.04	0.16	0.04	0.04	0.04
25	0.22	0.29	0.29	0.08	0.22	0.26	0.59	0.29

Discussion

For the balanced synthetic datasets, which contain attributes from different distributions, a smaller number of permutations implies that the permutation test overestimates the probability that the null hypothesis is true. Thus, fewer permutations are more likely to lead to false acceptance of the null hypothesis than to false rejection. However, the qualitative relationship between the probability and the number of data points appears to be preserved; thus, a small number of permutations is sufficient to make qualitative statements regarding their relationship. In general, at least 999 permutations should be selected for an effective approximation of the true probability according to Edgington [17] (see Supplementary section Comparison of Classification Accuracy to Chance Level). In line with classical statistics, we suggest a probability threshold of 5% that the null hypothesis is true—that is, to consider the result of a classification task to be significant. In this study, this value proved to be useful to interpret the results of the smallest and larger datasets. We used the same original and permuted datasets to analyse the characteristics of different metrics. The comparison of the dependence of the recall (Fig. 3) and the corresponding probability (Fig. 3) on the dataset size confirms that a high score alone does not imply a relationship between features and target variables. This is also evident in the only moderate increase of the MCC with a simultaneous strong decrease in the associated probability as well as in the narrowing of the confidence interval of all metrics when using more data points. A narrowing confidence interval implies that it becomes increasingly unlikely to obtain values much larger or much smaller than the mean. This is consistent with the

decreasing probability of the permutation test, whereas the score of the metric remains the same or even decreases. Thus, the score alone is not sufficient and a permutation test should be performed in any case. In addition, the result of the permutation test also depends on the size of the dataset.

The datasets studied are somewhat predictable, since they are generated with a small difference in the distribution of the features of both classes. In other words, the null hypothesis can be rejected for these data, and the metrics should provide the lowest possible probability for it. From the much lower probabilities obtained by performing a permutation test using the MCC compared to those obtained using the recall, it is evident that the MCC is better at detecting a relationship between the features and the target variable than the recall. This is also true for the F_1 score calculated from recall and precision. Cohen's κ, MCC, accuracy, and balanced accuracy are equally suitable for the evaluation of the studied datasets. However, one should keep in mind that previous research [27] considers MCC more suitable and accuracy rather unsuitable for unbalanced datasets, which are not examined here (see Supplementary Tables S1 Table, S2 Table). It should be noted that the high probabilities $> 5\%$ of the null hypothesis being true for all metrics are partially due to the small number of features used and the small difference in the value distributions of the two classes. With more features and a larger difference between the classes, better probabilities could be obtained with only a few data points.

Further, the fact that the averaged score of the nCVs in Fig. 4 is equal to the score of the rnCV is due to the fact that they are computed from the same predictions in the same manner. However, the probability of the nCVs is consistently higher than that of the rnCVs because they are calculated in a different manner: For the nCVs, the result of a single nCV of the original dataset is taken and compared to the results of the single nCVs of the permuted dataset to obtain an empirical probability. In this case, due to the variance of the nCVs, the score of the original dataset may be slightly underestimated and the score of the permutation may be slightly overestimated, thereby resulting in an overestimated probability. For the rnCV, the scores of the nCVs that constitute the rnCV are first averaged, and this averaged score is then compared with the scores of the permutations averaged in the same manner. This method reduces the variance of the results, thereby making it less likely that a permutation will randomly perform better or worse than the original dataset. Thus, there is an improvement in the reliability of the results of rnCV compared to those of nCV, which consequently reduces the obtained probability of the null hypothesis being true when the null hypothesis is in fact not true. Please note that this direct comparison is possible because the same permutations are used and the probability calculation for the rnCV utilises the results of the nCVs.

For the CESAR dataset, we found that feature selection reduces the F_1 score and Cohen's kappa as well as the probability that the null hypothesis is true for both metrics. This, again, reveals that the absolute value of the metric is highly dependent on the structure and size of the dataset and may be random. Thus, specifying the probability that the null hypothesis is true is particularly important for small datasets.

The score of the dataset with 50 data points improves only slightly for each of the metrics studied using feature selection when the number of data points is increased more than tenfold to 515 data points. This suggests that the dependency between labels and features is not sufficiently large to achieve better prediction quality and that 50 data points is close to the optimum.

With 50 permutations, used for the permutation test without feature selection, all metrics except F_1 score and recall attain the best possible probability for 515 data points. For 50 data points, the second best probability is achieved under the same conditions.

With feature selection, all metrics achieve the best possible probability for 25 permutations for 50 and 515 data points (see Table S4 Table). This indicates that 25 permutations are not sufficient to resolve the probability well enough to represent the differences between the metrics and between the datasets comprising 50 data points and 515 data points. The fact that the F_1 score is the only metric for 50 data points and the recall is the only metric for 515 data points that have a probability greater than 10% without feature selection and that they achieve the best possible probability with feature selection suggests that the estimator learns correlations during training on the unselected features that do not generalise. It also follows that it may be worthwhile to examine more than one metric, as they may happen to perform particularly well or poorly on the dataset.

From the improvement of the score by feature selection for most of the metrics on the full dataset and the dataset comprising 50 points, it can be concluded that only non-predictive or low-predictive attributes are eliminated, which were previously used by the classifier for prediction and, thus, worsened the predictivity.

Feature selection results in a slightly worse score for the 25 data points dataset for numerous metrics, but simultaneously reduces the probability of the null hypothesis being true, thereby suggesting that the features of the data structure—such as the imbalance between classes—led to the better score and not a better trained model. The reduction in the obtained probability of the null hypothesis being true must be even more significant than what is evident from the figures, since only half of the permutations of the evaluation without feature selection were used to calculate the probabilities for the evaluation with feature selection; thus slightly higher probabilities should actually be expected.

The ML estimator utilises the real dependency between features and labels in the data to predict, from the full dataset, the answers to the selected item 5b with less than 5% probability for the null hypothesis based on the answers to the other questions, with a MCC of 0.35; this is well above the expected value of zero of a random classification and particularly well above the lowest possible value of -1. The similar prediction quality on only 10% of the data suggests that the dependence between questions is not sufficiently large to achieve much better accuracy.

In conclusion, it is important to specify a probability value for the null hypothesis, particularly for small datasets and moderately good metrics. To obtain this probability, we suggest using a non-parametric permutation test in combination with a rnCV approach, which yields more reliable results than the traditional nCV method. Our results reveal that the probability determined by the permutation test is higher when the recall or F_1 score is used, compared to the MCC for our dataset, where a low probability is expected. Furthermore, we found that evaluating datasets with multiple metrics can help to better assess the predictive quality of the model, as revealed by the analysis of the CESAR dataset.

Supporting information

Training of the algorithm

Comparison of Classification Accuracy to Chance Level

Data generation for comparison of methods and metrics

Metrics

S1 Table. Overview of the characteristics of different metrics. In this table, the advantages and disadvantages of accuracy (ACC), balanced accuracy (BA), Cohen's

Kappa, and F_1 Score are presented.

S2 Table. Overview of the characteristics of different metrics. In this table, the advantages and disadvantages of precision/positive predictive value (PPV), recall, receiver operating characteristic curve (ROC) and Matthews correlation coefficient (MCC) are presented.

S1 Fig. (a) Dependence of the prediction score as well as (b) the probabilities of the associated permutation tests on the number of data points using MCC, Cohen's Kappa, accuracy, and balanced accuracy. The scores of MCC and Cohen's Kappa were normalised to the same range of values from 0 to 1 as the other two metrics. The coloured area represents the 95% confidence interval calculated by bootstraping over all datasets.

S3 Table. Scores with nested feature selection for repeated nested cross-validation for a randomly selected data subset.

S4 Table. Probabilities (in %) with nested feature selection for repeated nested cross-validation for a randomly selected data subset.

References

1. Bzdok D, Altman N, Krzywinski M. Statistics versus machine learning. Nature Methods. 2018;15(4):233–234. doi:10.1038/nmeth.4642.

2. Unsal S, Atas H, Albayrak M, Turhan K, Acar AC, Doğan T. Learning functional properties of proteins with language models. Nature Machine Intelligence. 2022;4(3):227–245. doi:10.1038/s42256-022-00457-9.

3. Jalota C, Agrawal R. Analysis of Educational Data Mining using Classification. Faridabad, India: IEEE; 2019. Available from: `https://ieeexplore.ieee.org/document/8862214/`.

4. AIX-COVNET, Roberts M, Driggs D, Thorpe M, Gilbey J, Yeung M, et al. Common pitfalls and recommendations for using machine learning to detect and prognosticate for COVID-19 using chest radiographs and CT scans. Nature Machine Intelligence. 2021;3(3):199–217. doi:10.1038/s42256-021-00307-0.

5. Vabalas A, Gowen E, Poliakoff E, Casson AJ. Machine learning algorithm validation with a limited sample size. PLOS ONE. 2019;14(11):e0224365. doi:10.1371/journal.pone.0224365.

6. Hilbert S, Coors S, Kraus E, Bischl B, Lindl A, Frei M, et al. Machine learning for the educational sciences. Review of Education. 2021;9(3). doi:10.1002/rev3.3310.

7. Géron A. Hands-on machine learning with Scikit-Learn, Keras, and TensorFlow: concepts, tools, and techniques to build intelligent systems. 2nd ed. Sebastopol, CA: O'Reilly Media, Inc; 2019.

8. Kohavi R. A Study of Cross-Validation and Bootstrap for Accuracy Estimation and Model Selection. IJCAI'95. San Francisco, CA, USA: Morgan Kaufmann Publishers Inc.; 1995.

9. Raudys SJ, Jain AK. Small sample size effects in statistical pattern recognition: recommendations for practitioners. IEEE Transactions on Pattern Analysis and Machine Intelligence. 1991;13(3):252–264. doi:10.1109/34.75512.

10. Bone D, Goodwin MS, Black MP, Lee CC, Audhkhasi K, Narayanan S. Applying machine learning to facilitate autism diagnostics: pitfalls and promises. Journal of Autism and Developmental Disorders. 2015;45(5):1121–1136. doi:10.1007/s10803-014-2268-6.

11. Kassraian-Fard P, Matthis C, Balsters JH, Maathuis MH, Wenderoth N. Promises, Pitfalls, and Basic Guidelines for Applying Machine Learning Classifiers to Psychiatric Imaging Data, with Autism as an Example. Frontiers in Psychiatry. 2016;7. doi:10.3389/fpsyt.2016.00177.

12. Krstajic D, Buturovic LJ, Leahy DE, Thomas S. Cross-validation pitfalls when selecting and assessing regression and classification models. Journal of Cheminformatics. 2014;6(1):10. doi:10.1186/1758-2946-6-10.

13. Combrisson E, Jerbi K. Exceeding chance level by chance: The caveat of theoretical chance levels in brain signal classification and statistical assessment of decoding accuracy. Journal of Neuroscience Methods. 2015;250:126–136. doi:https://doi.org/10.1016/j.jneumeth.2015.01.010.

14. Wasserstein RL, Lazar NA. The ASA Statement on p-Values: Context, Process, and Purpose. The American Statistician. 2016;70(2):129–133. doi:10.1080/00031305.2016.1154108.

15. Ojala M, Garriga GC. Permutation Tests for Studying Classifier Performance. 2009 Ninth IEEE International Conference on Data Mining. 2009; p. 908–913. doi:10.1109/ICDM.2009.108.

16. scikit learn. 3.1. Cross-validation: evaluating estimator performance; Retrieved April 20, 2022. Available from: `https://scikit-learn.org/stable/modules/cross_validation.html`.

17. Edgington ES. Approximate Randomization Tests. The Journal of Psychology. 1969;72(2):143–149. doi:10.1080/00223980.1969.10543491.

18. Chicco D, Warrens MJ, Jurman G. The Matthews Correlation Coefficient (MCC) is More Informative Than Cohen's Kappa and Brier Score in Binary Classification Assessment. IEEE Access. 2021;9:78368–78381. doi:10.1109/ACCESS.2021.3084050.

19. Kotsiantis S, Kanellopoulos D, Pintelas P. Handling imbalanced datasets: A review. GESTS International Transactions on Computer Science and Engineering. 2005;30:25–36.

20. Feurer M, Hutter F. Hyperparameter optimization. In: Hutter F, Kotthoff L, Vanschoren J, editors. Automated Machine Learning: Methods, Systems, Challenges. Cham: Springer International Publishing; 2019. p. 3–33. Available from: `https://doi.org/10.1007/978-3-030-05318-5_1`.

21. Chandrashekar G, Sahin F. A survey on feature selection methods. Computers & Electrical Engineering. 2014;40(1):16–28. doi:10.1016/j.compeleceng.2013.11.024.

22. Malone S, Küchemann S, Edelsbrunner PA, Lichtenberger A, Altmeyer K, Schumacher R, et al.. CESAR 0 data; 2021. Available from: `osf.io/p476u`.

23. Küchemann S, Malone S, Edelsbrunner P, Lichtenberger A, Stern E, Schumacher R, et al. Inventory for the assessment of representational competence of vector fields. Physical Review Physics Education Research. 2021;17(2):020126.

24. Questback GmbH. EFS Survey (EFS Winter 2018); 2019. Available from: `https://www.unipark.com/en/survey-software/`.

25. Pedregosa F, Varoquaux G, Gramfort A, Michel V, Thirion B, Grisel O, et al. Scikit-learn: Machine Learning in Python. Journal of Machine Learning Research. 2011;12:2825–2830.

26. Küchemann S, Malone S, Edelsbrunner P, Lichtenberger A, Stern E, Schumacher R, et al. Inventory for the assessment of representational competence of vector fields. Phys Rev Phys Educ Res. 2021;17:020126. doi:10.1103/PhysRevPhysEducRes.17.020126.

27. Chicco D, Jurman G. The advantages of the Matthews correlation coefficient (MCC) over F1 score and accuracy in binary classification evaluation. BMC Genomics. 2020;21(1):6. doi:10.1186/s12864-019-6413-7.

Figure belongs to manuscript file Click here to access/download;Figure;Fig 1.tiff

Fig 1.tiff

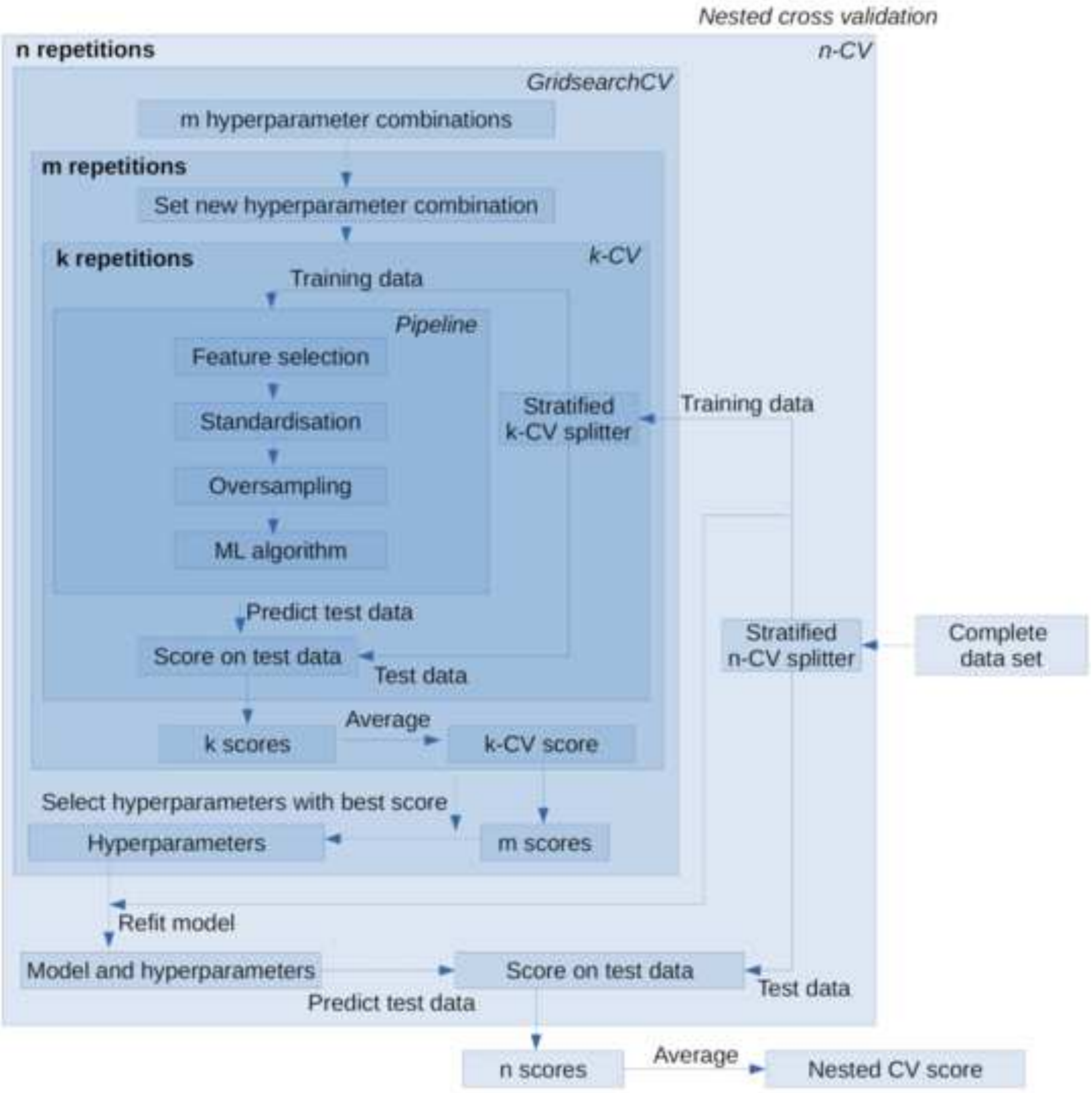

Figure belongs to manuscript file
Click here to access/download;Figure;Fig 2.tiff

Fig 2.tiff

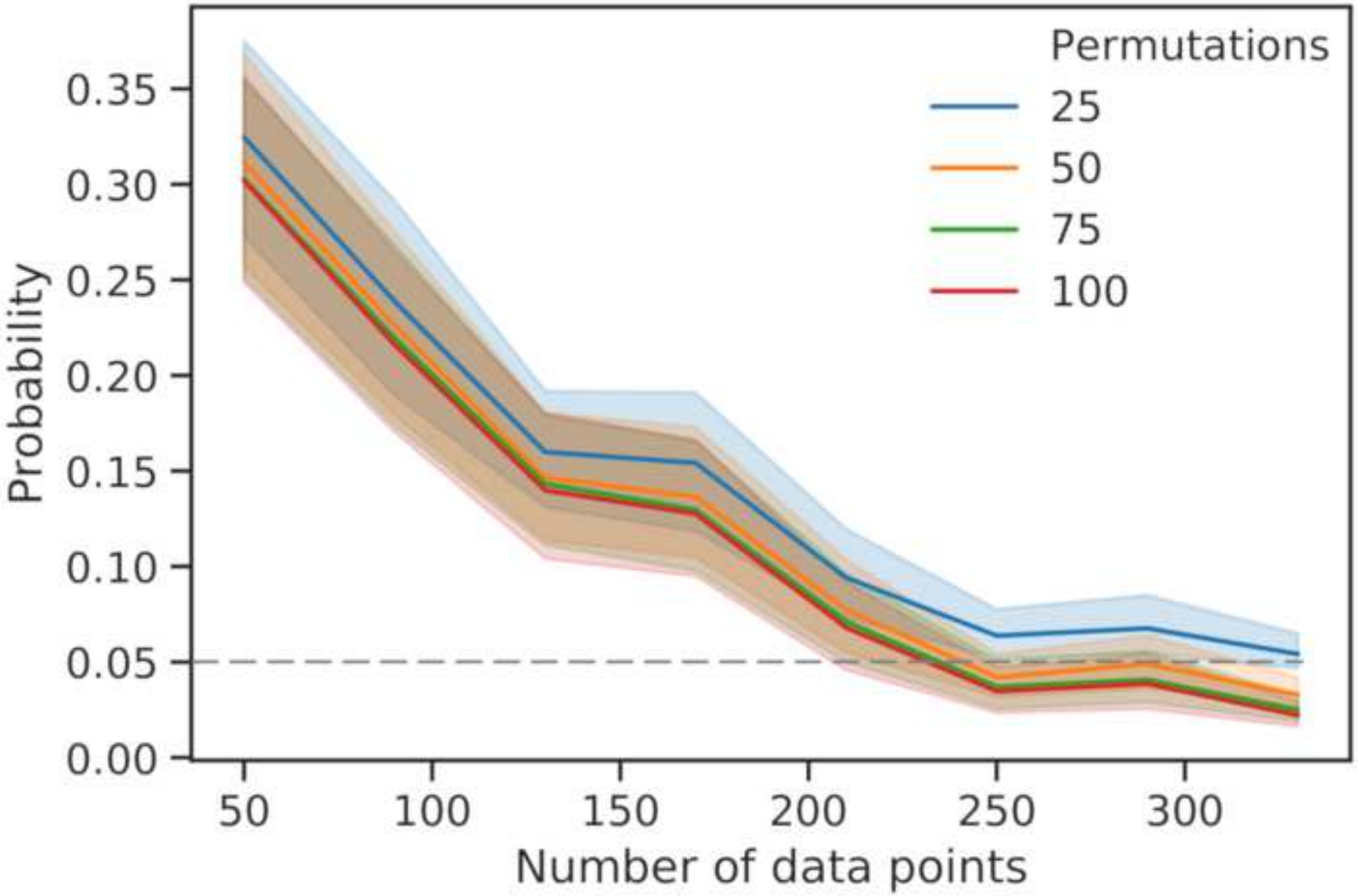

Figure belongs to manuscript file

Click here to access/download;Figure;Fig 3.tiff

Pasted Layer

(a)

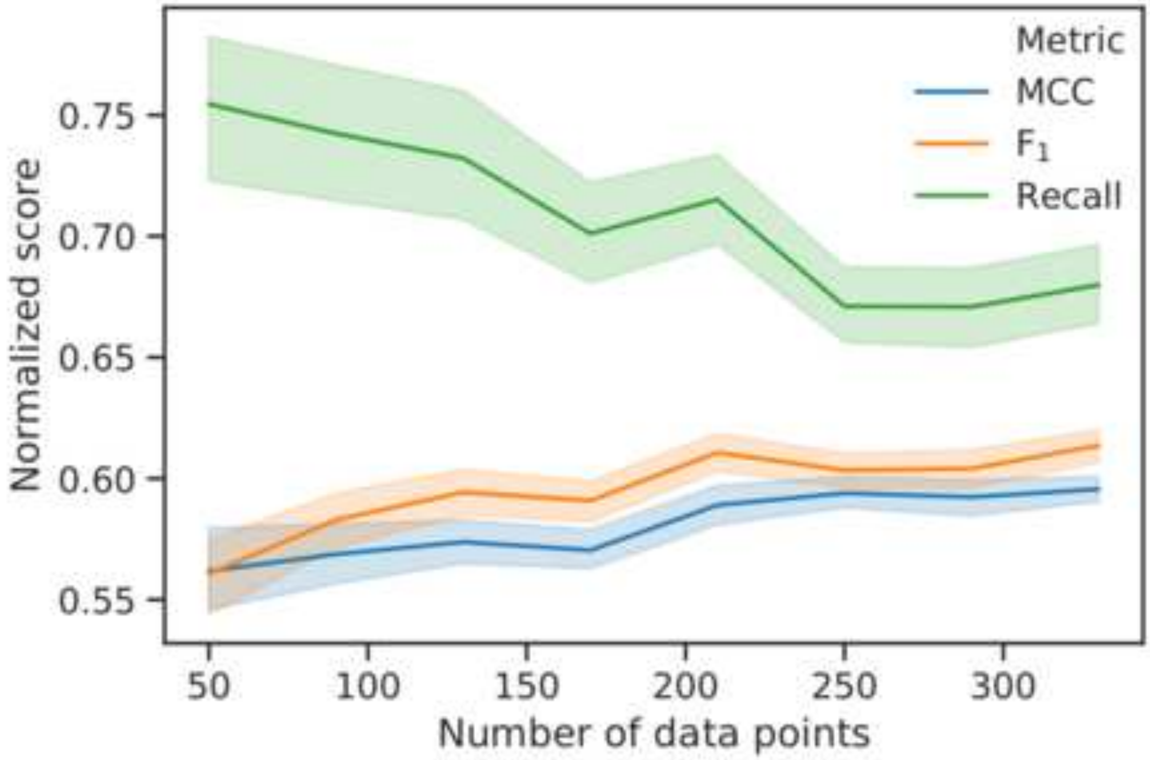

(b)

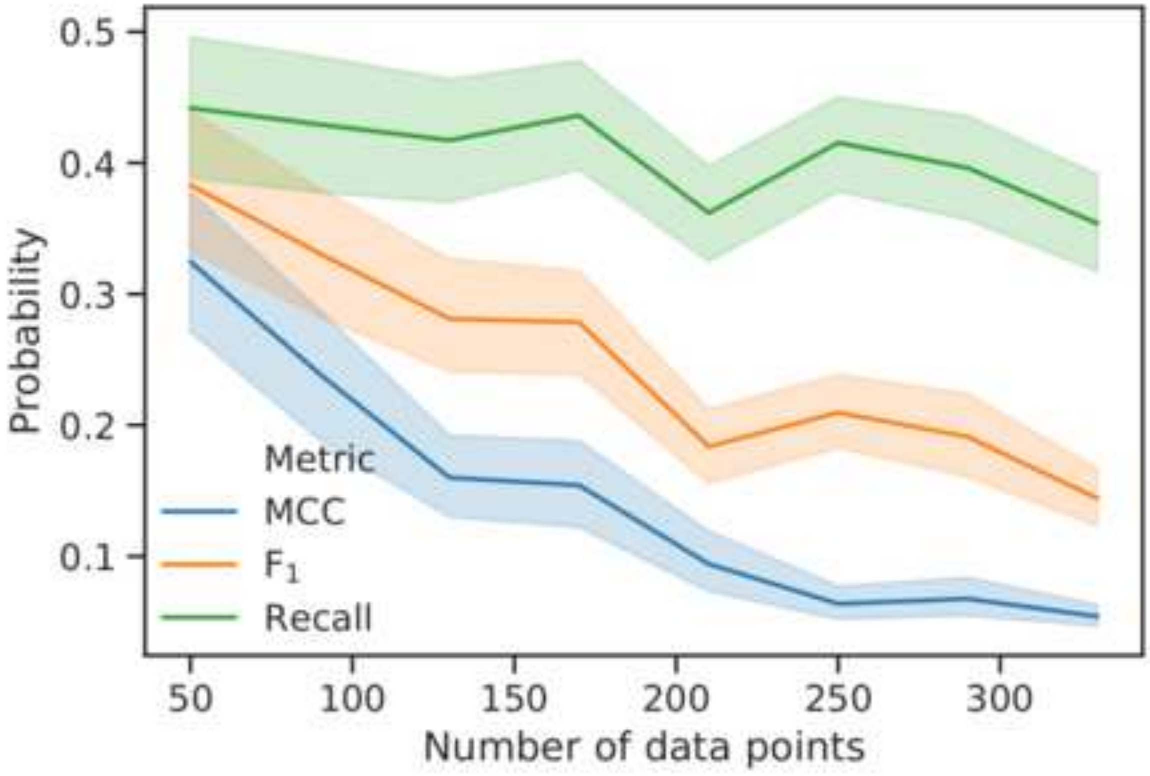

Figure belongs to manuscript file
Pasted Layer

Click here to access/download;Figure;Fig 4.tiff

(a)

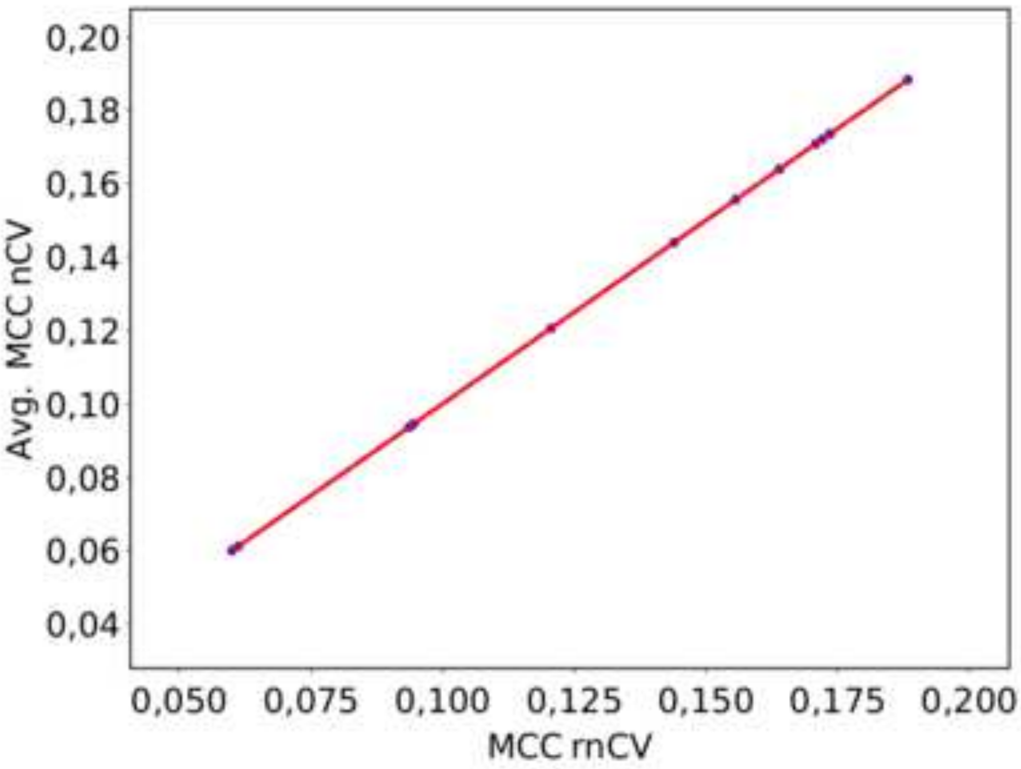

(b)

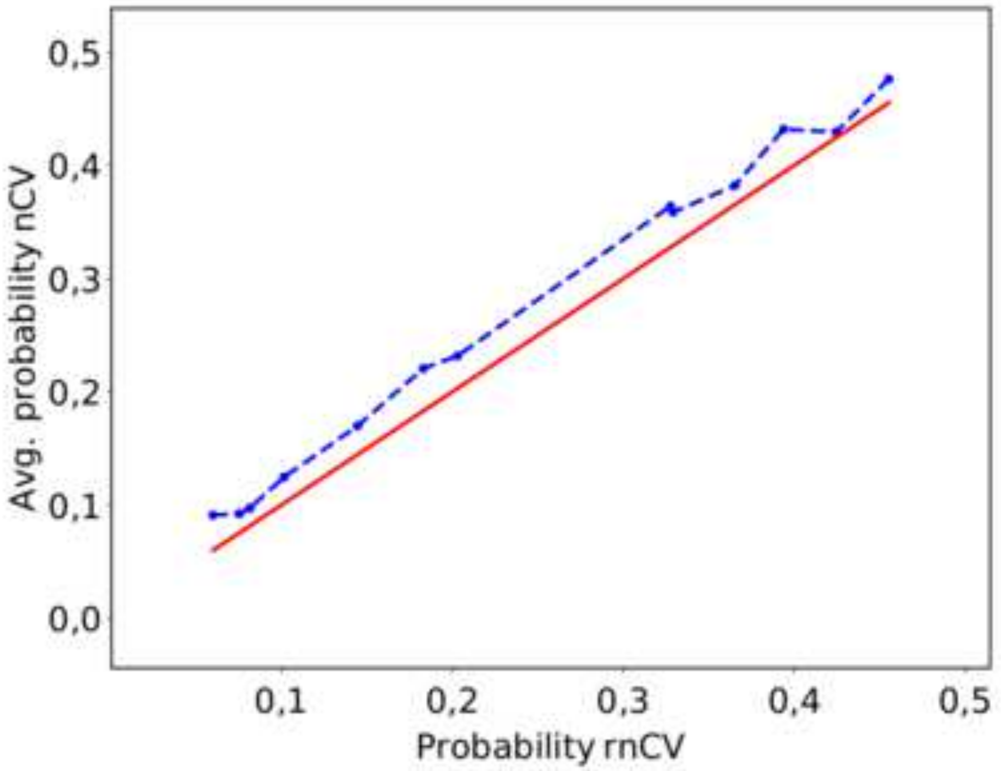

Supporting information

Click here to access/download
Supporting Information
supporting_information.pdf

Figure belongs to supporting_information.pdf file

Click here to access/download
Supporting Information
S1_Fig.tiff
